# Novel Drugs Development for Cardio-/Cerebrovascular Diseases

**DOI:** 10.1155/2014/467936

**Published:** 2014-06-17

**Authors:** Joen-Rong Sheu, Philip Aloysius Thomas, Juei-Tang Cheng

**Affiliations:** ^1^Graduate Institute of Medical Sciences, College of Medicine, Taipei Medical University, Taipei 110, Taiwan; ^2^Department of Microbiology, Institute of Ophthalmology, Joseph Eye Hospital, Tiruchirappalli, Tamil Nadu 620 001, India; ^3^Department of Medical Research, Chi-Mei Medical Center, Yung Kang City, Tainan 101, Taiwan

Cardiovascular and cerebrovascular diseases are the leading causes of death worldwide. According to the current epidemiological data, it is expected that, till 2020, coronary artery disease and cerebral hemorrhage are still the first and second causes of death of human beings, even though the order of the death causes due to human diseases would be changed significantly. Despite various advances in the understanding of the diseases, pharmacological treatment by conventional medicine has not obtained satisfactory results. However, recent studies suggest that natural products and traditional herbal medicine are a potential candidate for the preventative treatment of the disorders. Therefore, we have invited the researchers to contribute few research/review papers to provide solid evidence that supports the application of bioactives/traditional herbal medicine (THM) in prevention and treatment of cardiovascular and cerebrovascular diseases.

The first paper of this special issue investigates the role of peroxisome proliferator-activated receptors (PPAR*δ*) in ginseng-induced modification of cardiac contractility. It was suggested that ginseng could enhance cardiac contractility through increased PPAR*δ* expression in cardiac cells. The second paper investigates the role of musclin, a novel skeletal muscle-derived factor found in the signal sequence trap of mouse skeletal muscle cDNAs, using animal model of hypertension and characterizes its direct effect on vascular contraction. This paper provides the evidence that supports the fact that musclin is involved in hypertension and, thus, it is appropriate to consider as a novel target for treatment of hypertension, and another paper in this issue suggests that allantoin, as imidazoline I-1 receptors (I-1R) agonist, has the potential to develop as a new therapeutic agent for hypertension.

Amarogentin prevents platelet activation through the inhibition of PLC*γ*2-PKC cascade and MAPK pathway. This hypothesis is well presented in this special issue and findings suggest that amarogentin may offer therapeutic potential for preventing or treating thromboembolic disorders. Many clinical reports have suggested that the ascorbyl free radical (Asc^•^) can be treated as a noninvasive, reliable, real-time marker of oxidative stress, but its generation mechanisms in human blood have rarely been discussed. A paper in this issue studied upstream substances, enzyme inhibitors, and free radical scavengers to delineate the mechanisms of Asc^•^ formation in human platelet-rich plasma (PRP). This paper shows a well-defined protocol that adopts the hypothesis that Asc^•^ formation is associated with the inhibitors of NADPH oxidase (NOX), cyclooxygenase (COX), lipoxygenase (LOX), cytochrome P450 (CYP450), mitochondria complex III, nitric oxide synthase (NOS), and mitochondria in human PRP. Another subsequent paper shows that the cardioprotective effect of hypertonic saline is associated with inhibitory effect on macrophage migration inhibitory factor in sepsis; the authors of this paper suggest that hypertonic saline improves endotoxemia-induced myocardial contractility and prevents circulatory failure, contributing to the improvement of intracellular calcium handling process.

Another interesting paper in this special issue presents a pig model of myocardial ischemia/reperfusion (MIR) injury to investigate the maximum rate of change of left ventricular pressure, left ventricular end-diastolic pressure, and left intraventricular pressure. The role of *δ*-opioid receptor activation using D-Ala2, D-Leu5-enkephalin (DADLE) in both early (D1) and late (D2) phases of cardioprotection is identified in this paper and shows that DADLE after the ischemia has no benefit but combined treatment with anisodamine, a naturally occurring atropine-like compound, seems to have a marked postischemic cardioprotection. Therefore, this paper recommends that anisodamine is helpful in combination with DADLE for postischemic cardioprotection. Rutaecarpine (RUT), a major bioactive ingredient isolated from the Chinese herb* Evodia rutaecarpa*, possesses a wide spectrum of biological activities, including anti-inflammation and prevention of cardiovascular diseases. A derivative of this compound, bromo-dimethoxyrutaecarpine (Br-RUT), has been taken for a study in this special issue to evaluate its cardioprotective role. This paper establishes that Br-RUT has very low cytotoxicity in RAW 264.7 macrophages but retains its activities against inflammation and vasodilation via enhanced expression of transient receptor potential vanilloid type 1 and activated endothelial nitric oxide synthase (NOS) that could be beneficial for cardiovascular disease therapeutics.

We anticipate that readers will find that this special issue reports the important contests, prospects, and existing advances that are presently being oppressed in cardiovascular research with the potential to encourage the application of novel drug development for elevating the attention and treatment of patients with cardiovascular disease.

*Joen-Rong Sheu* *Philip Aloysius Thomas* *Juei-Tang Cheng*

